# Selection and application of methanol-utilizing bacteria from tomato leaves for biocontrol of gray mold

**DOI:** 10.3389/fmicb.2024.1455699

**Published:** 2024-10-18

**Authors:** Hiroyuki Suenaga, Tomoko Hira, Takahiro Yoshimura, Takuji Oka, Daisuke Hira

**Affiliations:** ^1^Department of Biotechnology and Life Sciences, Faculty of Biotechnology and Life Sciences, Sojo University, Kumamoto, Japan; ^2^Hira Labo, Kumamoto, Japan; ^3^Yoshimura Pharm, Kumamoto, Japan

**Keywords:** tomato, *Botrytis cinerea*, methylotroph, *Serratia rubidaea*, gray mold, biocontrol, microbial communities

## Abstract

Gray mold, caused by *Botrytis cinerea*, is a significant threat to tomato production. Traditional chemical control methods have become increasingly ineffective because of the development of resistance. This study aimed to isolate methanol-utilizing bacteria from tomato leaves and evaluate their biocontrol potential against gray mold. To obtain bacterial suspensions, tomato leaf samples were collected and washed. We analyzed the microbial communities of these samples using 16S rRNA amplicon sequencing and identified several methylotrophic strains. Among these, 405 isolated strains were cultivated on a solid low-nutrient inorganic salt medium containing methanol, and 7 strains exhibiting considerable antifungal activity against *B. cinerea* were identified. Greenhouse tests revealed that two strains—SY163 and SY183—significantly reduced the severity of gray mold on tomato leaves. Disease index scores and the area under the disease progress curve values confirmed the efficacy of these strains as biocontrol agents. Statistical analysis indicated the effectiveness of pre- and co-application of these strains with *B. cinerea*. Phylogenetic analysis identified *Serratia rubidaea* as the inhibitory strain. The biocontrol activity is likely mediated through the production of antifungal compounds and suppression of *B. cinerea* sporulation. This study provides the basis for developing a technology of gray mold suppression by controlling the abundance of *S. rubidaea* in plant microbial communities.

## 1 Introduction

Pathogens that cause significant damage to crop plants include fungi, bacteria, viruses, and nematodes. Crop losses pose a major threat to food production, with approximately 27–42% of the global food production being lost to plant diseases arising from these pathogens. Various biotic and abiotic factors contribute to economic yield losses in crops, with diseases caused by filamentous fungi being the most important factor (Singh, 2014).

*Botrytis cinerea*, the causal agent of gray mold, is considered an important pathogen worldwide. Although global expenditures related to the implementation of control measures against *B. cinerea* exceed €1 billion per year, product and quality losses are a major concern (Dean et al., 2012). Even in tomato, which accounts for approximately 16% of total vegetable production, gray mold is one of the most destructive rot diseases, posing a serious threat to its yield (Food and Agriculture Organization of the United Nations, 2023; Liu et al., 2021). The host can be infected by fungal mycelia, conidia, or sclerotia. Additionally, *B. cinerea* has a short life cycle and exhibits rapid genetic variation, complicating the management of gray mold in agricultural production (Breeze, 2019). Although benzimidazoles, dicarboximide fungicides, and anilinopyrimidine fungicides (Banno et al., 2008; Leroux et al., 1999; Leroux, 2007) have been used for *B. cinerea* control, resistance to these fungicides remains a challenge. Owing to its high genetic variability, *B. cinerea* is associated with an increased risk of fungicide resistance. In many countries, the long-term and frequent use of these fungicides has led to the development of *B. cinerea* resistance (Esterio et al., 2017; Liu et al., 2021).

The most common agents for the biocontrol of *B. cinerea* include filamentous fungi of the genera *Trichoderma*, *Ulocladium*, and *Gliocladium*; bacteria of the genera *Bacillus* and *Pseudomonas*; and yeasts of the genera *Pichia* and *Candida* (Elmer and Michailides, 2007); among them, *Trichoderma* fungi have been widely studied (Vinale et al., 2008; Dutta et al., 2023; Vos et al., 2015). Biocontrol is an alternative and environmentally friendly strategy for controlling plant diseases and reducing chemical use in agriculture. In addition to its biocontrol activity, *Trichoderma* is widely used for plant growth promotion (PGP) through the regulation of phytohormone formulation. Several studies have isolated *Trichoderma* strains from the rhizosphere and indicated their ability to control disease-causing bacteria (Vinale et al., 2008; Hajji-Hedfi et al., 2023). A previous study isolated *Trichoderma* strains from cucumber leaves and evaluated their ability to control gray mold in various plants (Sawant, 2014).

Most studies on plant growth-promoting bacteria (PGPB) have focused on their ability to enhance plant growth and suppress plant pathogens. For example, *Bacillus* and *Pseudomonas* species exhibit antagonistic activity against a wide range of plant pathogens through the production of antibiotics, enzymes, and other bioactive compounds (Kloepper et al., 2004; Lugtenberg and Kamilova, 2009). Similarly, the potential of *Rhizobium* and *Azospirillum* species in promoting plant growth and inducing systemic resistance against pathogens has been reported (Bashan and de-Bashan, 2010; Zamioudis and Pieterse, 2012). These PGPBs enhance plant health by improving nutrient uptake, producing phytohormones, and stimulating the plant’s immune system.

Methylotrophs are microorganisms that use one-carbon (C1) compounds, such as methanol and methylamine, as carbon sources. They settle on the leaves of some plants that release methanol, which is used as a carbon source for growth. Thus, a symbiotic relationship exists in which methylotrophs produce plant hormones that benefit the plants (Iguchi et al., 2015; Omer et al., 2004; Trotsenko et al., 2001). Methylotrophic bacteria can survive in various extreme environments, including low and high temperatures and drought conditions (Romanovskaia et al., 2005; Sapp et al., 2018; Amin et al., 2017; Kerry et al., 2018). Plants develop stress tolerance by receiving valuable nutrients from methylotrophs under stress. Additionally, PGP methylotrophic microbiota help plants grow and adapt to adverse environmental conditions. The rhizosphere, epiphytic, and endophytic microbiomes play an important role in plant growth and adaptation (Kumar et al., 2019). However, to the best of our knowledge, no studies have reported the use of C1-utilizing bacteria for controlling gray mold.

In this study, we collected symbiotic methylotrophic bacteria from plant leaves and searched for microorganisms that can control *B. cinerea*. We evaluated the ability of the identified microbes to control *B. cinerea* on plant foliage.

## 2 Materials and methods

### 2.1 Biomaterials

*B. cinerea* (Agricultural Biological Resources Gene bank MAFF No. 241680 Strain), a known cause of gray mold on tomato plants, was used in this study. Tomato leaves and stems (Reiki; Sakata Seed Corporation, Kanagawa, Japan) sampled for microbial collection were grown in plastic greenhouses at Yoshimura Farm (130°38′21.68′′E, 32°43′42.599′′N, Kumamoto, Japan). Grafted tomato saplings (Momotaro Haruka) were purchased from Kokkaen (Osaka, Japan) for use in biological control bioassays.

### 2.2 Buffer and medium composition

Phosphate-buffered saline (PBS), comprising 8.0 g NaCl, 0.2 g KCl, 1.15 g Na_2_HPO_4_, and 0.2 g KH_2_PO_4_ dissolved in 1.0 L distilled water, was prepared and used for the recovery of microbes from tomato leaf and stem surfaces.

The composition of the inorganic salt liquid medium containing methanol was as follows: 2.0 g KH_2_PO_4_, 2.4 g K_2_HPO_4_, 1.0 g (NH_4_)_2_SO_4_, 1.0 g KNO_3_, 0.2 g MgSO_4_, 1.0 g trace element mixture, 5 mL methanol, and 1.0 L distilled water (Meiberg and Harder, 1978; Aida and Nomoto, 1988). The medium composition was modified from that of a previously reported medium. The carbon source (methanol) was mixed to 0.5%. The inorganic solid medium containing methanol was prepared in 90- mm diameter petri dishes by adding 10.0 g gellan gum and 0.4 μg tunicamycin to the liquid medium. All components except for methanol and tunicamycin were dissolved in water and autoclaved at 120°C for 20 min. Methanol and tunicamycin were added to the medium via filter sterilization after autoclaving.

The nutrient broth (NB) liquid medium was prepared by dissolving NB (3.0 g beef extract, 5.0 g peptone) in 1.0 L of distilled water. The mixture was autoclaved at 120°C for 20 min. NB liquid medium was dispensed into test tubes.

Potato dextrose agar (PDA) medium was prepared by dissolving 4.0 g potato infusion powder (Sigma, 52424), 20.0 g glucose, and 20.0 g agar in 1.0 L of distilled water. The mixture was autoclaved at 120°C for 20 min. PDA medium was dispensed into petri dishes (90 mm diameter).

### 2.3 Sampling

In the plastic greenhouses, leaves and stems of 10 tomato plants (about 1 kg) per point were randomly collected from three points and stored them in polyethylene bags on July 1, 2020. All collected samples were transported to the laboratory of Sojo University (Kumamoto, Japan). In the laboratory, within 3 h of sampling, approximately 1 kg of tomato leaves and stems was distributed into three polyethylene bags, and 50 mL of PBS was added to each polyethylene bag using a Terumo syringe (10 mL) and CA sterile syringe filter (0.22 μm). The polyethylene bags containing leaves, stems, and wash buffer were shaken well by hand and allowed to stand at room temperature for 1 h. Approximately 20 mL of each washed suspension was collected in a 50-mL tube.

### 2.4 Analysis of microbial community composition

Washed suspensions were collected from tomato leaves and stems (approximately 20 mL each) and 0.5 mL of each of this suspension was dispensed into two Eppendorf tubes, resulting in six suspensions. Microbial DNA was extracted from tomato leaf and stem samples using ISOIL (NIPPON GENE, Tokyo, Japan) in accordance with the manufacturer’s protocol. In each sample, the V3–V4 hypervariable region of the 16S rDNA gene was amplified with an appropriate primer pair (U341F [5′-CCTACGGGRSGCAGCAG-3′] and 806R [5′-GGACTACHVGGGTATCTAAT-3′]) (Hansen et al., 1998; Walters et al., 2011). Polymerase chain reaction (PCR) was performed under the following conditions: an initial denaturation step at 94°C for 2 min; followed by 25 cycles of denaturation at 94°C for 15 s, annealing at 48°C for 30 s, and extension at 68°C for 30 s; and a final extension step at 68°C for 1 min. The PCR products of microbial 16S rRNA gene fragments were purified using Agencourt AMPure XP (Beckman Coulter, Brea, CA, United States). Tags unique to each sample were attached to the reverse primer for multiplexing. The PCR products were then analyzed using a MiSeq next-generation sequencer (Illumina, San Diego, CA, United States) at Fasmac (Kanagawa, Japan), which targeted the V3–V4 hypervariable region of the microbial 16S rRNA gene.

The raw paired-end FASTQ reads were demultiplexed using the FASTQ barcode splitter and imported into the Quantitative Insights Into Microbial Ecology 2 program (QIIME2, ver. 2019.10)^1^ (Bolyen et al., 2019). The demultiplexed reads were quality-filtered, denoised, chimera-checked, and dereplicated using the denoise-paired command from q2-dada2 plugin (Callahan et al., 2016). Taxonomic assignments were performed using the classify-sklearn command from q2-feature-classifier using a trained Naive Bayes classifier from SILVA database (release132).^2^ Reads identified as sequences from mitochondria or chloroplasts were excluded from the analysis using filter-table command from the q2-taxa plugin. Next, the align-to-tree-mafft-fasttree pipeline from the q2-phylogeny plugin was used to perform multiple sequence alignment, remove phylogenetically uninformative or ambiguously aligned sequences, and construct unrooted and rooted phylogenetic trees (Price et al., 2010; Katoh and Daron, 2013).

### 2.5 Data visualization and statistical analyses of microbial community composition

Data visualizations and statistical analyses of microbial community composition were performed using R (v4.3.1).^3^ QIIME2 output data were converted to a phyloseq object using the qza_to_phyloseq function using the qiime2R package (Bisanz, 2018). The phyloseq object was used to perform the following visualizations and analyses process in phyloseq (McMurdie and Holmes, 2013), tidyverse (Wickham et al., 2019), plotly (Sievert et al., 2024), and RColorBrewer (Neuwirth, 2022) packages. According to known taxonomic groups, bar charts of relative abundance at phylum and genus levels were constructed for tomato leaf and stem samples. The diversity of microbial communities within and between these samples was characterized using alpha and beta diversities, respectively. Alpha diversity was assessed based on observed operational taxonomic units (OTUs); abundance-based coverage estimator (ACE); and Chao 1, Shannon, and Simpson diversity indices. Beta diversity was assessed via nonmetric multidimensional scaling (NMDS, Bray–Curtis similarity matrix) based on microbial community structures.

### 2.6 Culture and isolation of methylotroph

The suspension samples were used to prepare serial dilutions of 1:5 (5-fold dilution), 1:25, 1:125, 1:625, 1:3125, and 1:15625. The inorganic salt medium plates containing methanol were coated with 50 μL of seven different suspension samples at different dilutions using a sterile Drigalski spatula. These plates were incubated at 30°C, and colony formation was observed daily. After colony formation was confirmed, the isolates were selected based on morphological characteristics, and each colony was collected using a platinum loop. The selection of similar colonies that occurred on the plate at the same time was avoided. Colonies were collected using the platinum loop and cultured in an inorganic salt liquid medium containing methanol and NB liquid medium. NB medium was used because some methylotrophs prefer heterotrophic and substrate-rich conditions. Two types of culture were performed: static culture (30°C) and shaking culture (150 rpm, 30°C). For example, in methylotrophic denitrifying bacteria, anaerobic nitrate respiration is promoted by static culture. Because some methylotrophic bacteria tend to grow aerobically and some anaerobically, they were cultured under both static and shaking conditions to ensure that they were comprehensively picked up. The cultures in which the suspension was confirmed were stored in glycerol at −60°C in the laboratory of Sojo University.

### 2.7 Screening of inhibitory bacterial strains

A 25-μL *B. cinerea* suspension (4.2 × 10^5^ conidia/mL) was applied to PDA plates (90 mm diameter), and the plates were incubated at 25°C. The time taken for *B. cinerea* mycelia to cover the plates was measured.

The plates coated with *B. cinerea* suspension were modified by drilling 2–8 holes (9 mm diameter) around the center of the plate. The holes were equidistant from each other and located 14 mm away from the edge of the plate. Then, 50 μL of each strain suspension (2 × 10^6^ CFU/mL) was placed in each hole and incubated at 25°C for 10 days.

The growth inhibitors of *B. cinerea* were examined using a dual plate assay. Agar blocks (5 mm × 5 mm) were cut from PDA plates on which *B. cinerea* had grown; these blocks were inoculated onto another PDA plate. Then, 1.0 μL of the suspension of the inhibitory strain (2 × 10^6^ CFU/mL) was dropped onto the edge of the plate and incubated statically at 25°C.

### 2.8 Identification of inhibitory bacterial strains

The V3–V4 region of the 16S rRNA gene from each inhibitory strain was approximately 400–450 bp in length. This region was amplified using the primer pair of U341F and 806R. The *gyrB* gene from each inhibitory strain was approximately 1,100 bp in length and was amplified using the primer pairs gyrF (5′-GAAGTCATCATGACCGTTCTGCATCGCTCAGGGTCAGGGTCAGAAAGTTTCGA-3′) and gyrR (5′-AGCAGGTACGATGTGCGAGCCAGTCTCAGACAGTCTCAGGCAGTCTCAGGTAT-3′) (Govil et al., 2021). In both reactions, for each inhibitory strain, the PCR mixture (50 μL) containing 1.0 μL of genomic DNA, 5.0 μL of 10× PCR Buffer for KOD-Plus-Neo, 5.0 μL of 2 mM dNTPs, 3.0 μL of 25 mM MgSO_4_, 1.0 μL of KOD-Plus-Neo, 1.5 μL of 10 μM forward primer, 1.5 μL of 10 μM reverse primer, and 33 μL of PCR-grade water (Promega) was placed in a microcentrifuge tube. PCR for 16S rRNA was conducted using the following thermocycling parameters: initial denaturation at 94°C for 2 min, followed by 25 cycles of denaturation at 98°C for 10 s, annealing at 50°C for 30 s, and extension at 68°C for 20 s. PCR for *gyrB* was conducted using the following conditions: initial denaturation at 94°C for 2 min, 25 cycles of denaturation at 98°C for 10 s, annealing at 50°C for 30 s, and extension at 68°C for 35 s. PCR products were visualized under UV light after standard ethidium bromide gel electrophoresis. The amplified products were purified using PCR Clean-UP System (Promega, USA) in accordance with the manufacturer’s protocol. The purified PCR products were stored at −20°C.

The PCR products were then separated using agarose gel electrophoresis and cloned into TOPO-Blunt (Invitrogen, Carlsbad, CA). Further, these products were cloned into the pCR Blunt II plasmid using Zero Blunt TOPO PCR cloning kit in accordance with the manufacturer’s protocol. The amplified PCR fragments were sequenced. Homology searches were performed via the NCBI BLAST database (Johnson et al., 2008) using the identified DNA sequences as queries, and molecular phylogenetic analysis was performed using 16S rRNA and *gyrB* genes. Alignment and phylogenetic tree estimation were performed using MAFFT 6.861 and RAxML 8.2.11 in ETE v3.1.2 (Huerta-Cepas et al., 2016), and the results were presented using iTOL (Letunic and Bork, 2021).

### 2.9 Biological control assays in plants

Strains that were highly effective in inhibiting the growth of *B. cinerea* in petri dish tests were examined for their ability to inhibit gray mold caused by *B. cinerea* in plants.

Biological control assays were performed using five tomato plants (2 months old) in each group as a normal group, a control group, and three biological control condition groups (BC1-BC3). Normal group: normal growth (no fungus application); control group: only *B. cinerea* applied on day 0; condition BC1: *B. cinerea* and each inhibitory strain applied on day 0; condition BC2: each inhibitory strain applied on the day before day 0 and *B. cinerea* and each inhibitory strain applied on day 0; and condition BC3: *B. cinerea* and each inhibitory strain applied on days 0 and each inhibitory strain applied on day 1. *B. cinerea* conidia suspension (4.2 × 10^5^ conidia/mL, 10 μL per leaf) was applied using a pipette. The inhibitory strains were SY163 and SY183, and an inhibitory strain culture suspension (2 × 10^6^ CFU/mL, 10 μL per leaf) was applied using a pipette.

All groups were allowed to grow at room temperature (18°C–22°C) for 22 days. During the biological control assay, 20 mL/pot of water was applied to the soil surface in the morning using a Falcon tube. The disease index of gray mold on each assessed tomato leaf was recorded daily, and the disease severity for each leaf was calculated. The disease index was expressed as a percentage of diseased leaf area and evaluated on a scale of 0–3 (0 = no disease symptoms, 1 = 0.1–24.9%, 2 = 25–49.9%, 3 = 50–100%) (Konishi et al., 2010). Disease severity was calculated using the following formula: {*Σ*(disease index × number of leaves by disease index)/(number of leaves assessed)}. In addition, conidia formation after 22 days was evaluated in all assessed leaves. The percentage of leaves with conidia formation was calculated for each plant using the following formula: (number of leaves that formed conidia)/(number of leaves assessed) × 100 (%), and the mean and standard error of the percentage of conidia formation were determined for each condition.

### 2.10 Statistical analyses of biological control

The area under the disease progression curve (AUDPC) was calculated using the aupdc function in the package agricolae (v.1.3–5) (De Mendiburu and Reinhard, 2015) in R (v.4.3.1) (R).^4^ The Dunnett test, which was used to compare the AUDPC of conditions BC1-BC3 against that of control group, was performed using the glht function in the package multcomp (v.1.4–25) (Bretz et al., 2010) in R.

The progression of gray mold over days was evaluated for different conditions and individuals (ID), and comparisons were performed between conditions. Linear mixed models were performed using the lmer function of the package lme4 (v.1.1–35.3) (Bates et al., 2024) in R. The mean values for each condition estimated from the model were calculated using the emmeans function in the package emmeans (v.1.10.2) (Lenth et al., 2024). The obtained mean values for each condition were compared via the contrast function using Dunnett’s post-hoc test.

## 3 Results

### 3.1 Microbial community analysis of tomato leaf and stem samples

**Figure 1:**
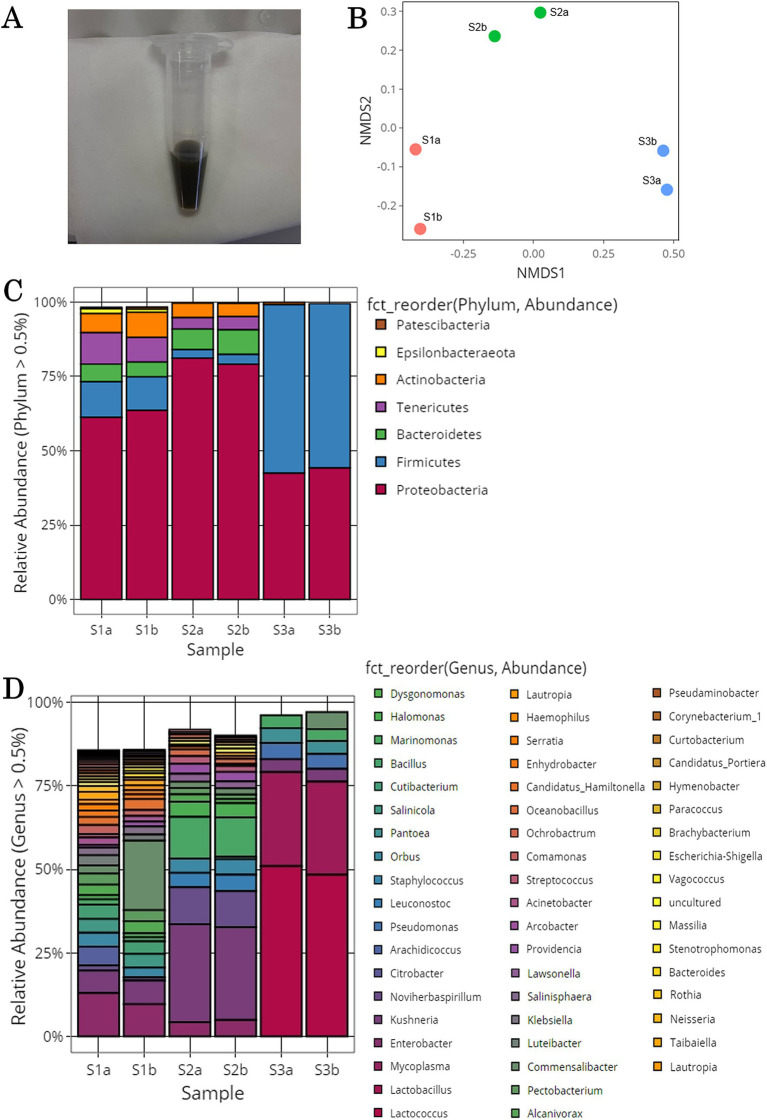
Microorganisms acquired from the surface of tomato leaf and stem. **(A)** Solution after washing the surface of the tomato leaves and stems. **(B)** Nonmetric multidimensional scaling (NMDS) plots generated from a matrix of Bray–Curtis dissimilarity values. **(C)** Microbial community structure at the phylum level. Bar plot showing the relative abundances of the seven dominant phyla (>0.5%). **(D)** Microbial community structure at the genus level. Bar plot showing the relative abundances of the 54 dominant genera (>0.5%).

Samples of tomato leaves and stems were collected from three different locations in the greenhouse and washed with PBS to obtain a gray suspension (Figure 1A). Each of the three samples (S1, S2, and S3) was divided into two parts (a and b), yielding six samples in total (S1a, S1b, S2a, S2b, S3a, and S3b). These six samples were sequenced using 16S rRNA amplicon sequencing. After sequence processing and quality control, a total of 254,333 bacterial reads were generated. The number of reads in each sample ranged from 102,195 for S3b to 12,354 for S2b (Supplementary Table S1).

To determine the alpha diversity within each sample (number of OTUs observed and Chao1, Shannon, and Simpson indices), two samples derived from the same origin (e.g., S1a and S1b) were analyzed. The results revealed similar values for species richness and evenness indices between S1a and S1b; however, differences were observed between samples with different origins (Supplementary Table S1). S1-derived samples (S1a and S1b) had high OTUs, ACE values, and Chao1 indices, indicating high species richness. In contrast, S3-derived samples (S3a and S3b) had low species richness. Moreover, S1-derived samples had higher Shannon and Simpson indices, suggesting a diverse microbial community with almost equal proportions of species. Conversely, S3-derived samples had lower Shannon and Simpson indices, indicating lower diversity and the potential for certain species to be significantly more abundant than others. Based on alpha diversity results, the species richness and diversity of the microbial communities on the surface of tomato leaf and stem samples may be dependent on the collection site.

The beta diversity between these six samples was examined using NMDS plots generated from a matrix of Bray–Curtis dissimilarity values (Figure 1B) and the microbial community structure (Figures 1C,D). In the NMDS plot, samples derived from the same origin were clustered together, whereas those derived from different origins were located away from each other. This suggests that similar to alpha diversity results, the microbial community of tomato leaves and stems tends to vary by sampling location. The microbial community analysis of all samples revealed seven phyla with relative abundances higher than 0.5% (Figure 1C). At the phylum level, *Proteobacteria* had the highest relative abundance across all samples. In S1a, S1b, S3a, and S3b, *Firmicutes* was the second most prevalent phylum; in S2a and S2b, Bacteroidetes was the second most prevalent phylum. The microbial community analysis of all samples revealed 54 genera with relative abundances higher than 0.5% (Figure 1D). At the genus level, *Lactococcus* and *Lactobacillus* were abundant in S3a and S3b; *Enterobacter* and *Pantoea* were abundant in S2a and S2b; and *Mycoplasma*, *Marinomonas*, and *Enterobacter* were abundant in S1a and S1b. Although microbial community structures of samples from the same origin were similar, differences were observed between samples derived from different origins. This finding was consistent with that of alpha and beta diversity.

**Figure 2:**
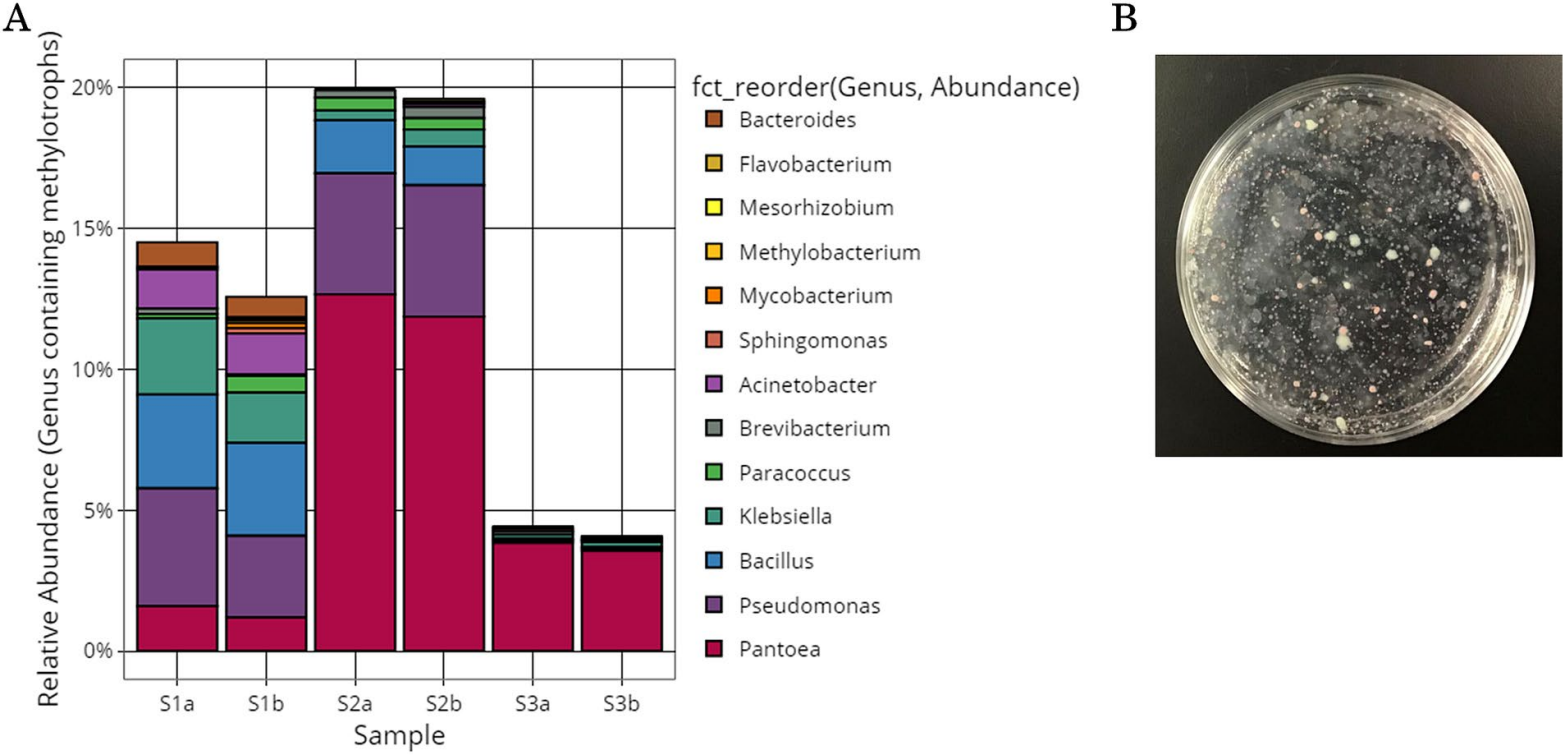
Methylotrophic bacteria isolated from the surface of tomato leaves and stems. **(A)** Microbial community structure of the genera containing methylotrophic bacterial strains. **(B)** Low-nutrient inorganic salt medium plate containing methanol cultures of tomato leaf and stem surface samples yielded colonies of various colors and sizes.

The possible extent of methylotroph on tomato leaf and stem surfaces was investigated. Previous studies have shown that methylotrophic bacterial strains exist in 83 genera, including *Pseudomonas*, *Methylobacterium*, and *Methylococcus* (Kolb, 2009; Kumar et al., 2016; Kumar et al., 2019; Gamit and Amaresan, 2023). In this study, 13 of these genera were identified: *Pantoea*, *Pseudomonas*, *Bacillus*, *Klebsiella*, *Acinetobacter*, *Bacteroides*, *Paracoccus*, *Brevibacterium*, *Sphingomonas*, *Mycobacterium*, *Methylobacterium*, *Flavobacterium*, and *Mesorhizobium*. The relative abundances of each genus in each sample are shown in Figure 2A and Supplementary Table S2. In all the samples, *Pantoea*, *Pseudomonas*, *Bacillus*, *Klebsiella*, and *Acinetobactor* had high relative abundances. For the overall microflora, the relative abundances were similar in the samples collected from the same location, but varied among sampling locations.

### 3.2 Isolation of methylotrophs from tomato leaf and stem samples

Methylotrophs were collected from the gray suspensions obtained from tomato leaf and stem surfaces. The gray suspension was placed on a low-nutrient inorganic salt solid medium plate containing methanol. After 6–14 days, colonies of various colors (red, yellow, and white) and sizes were obtained (Figure 2B). Colonies were picked from the inorganic salt solid medium plate containing methanol and cultured in inorganic salt liquid medium containing methanol or NB liquid medium. Culture samples of isolated strains were collected after 1–14 days of static culture and 1–13 days of shaking culture. In total, 405 isolated strains were obtained and frozen in glycerol. These isolated strains could grow under conditions in which methanol was the only carbon source. Plant leaves are inhabited by methylotrophic bacteria that utilize methanol, such as *Methylobacterium*, *Methylophilus*, *Methylibium*, and *Hyphomicrobium* (Iguchi et al., 2015; Lopez-Velasco et al., 2011).

### 3.3 Selection and identification of inhibitory bacterial strains

*B. cinerea* conidia were incubated on PDA plates at 25°C, and it was visually confirmed that the mycelia of *B. cinerea* had covered the plates on the 10th day (Supplementary Figure S1A). In a previous study, *B. cinerea* conidia were applied onto PDA plates and incubated at 22°C for 7 days, and similar mycelial conditions were observed (Vidal et al., 2020). Consistent with the results presented in Supplementary Figures S1B,C, the mycelium of *B. cinerea* was characterized by splitting, gray, branched, tree-like structures with numerous branching hyphae (Sultana et al., 2020).

**Figure 3:**
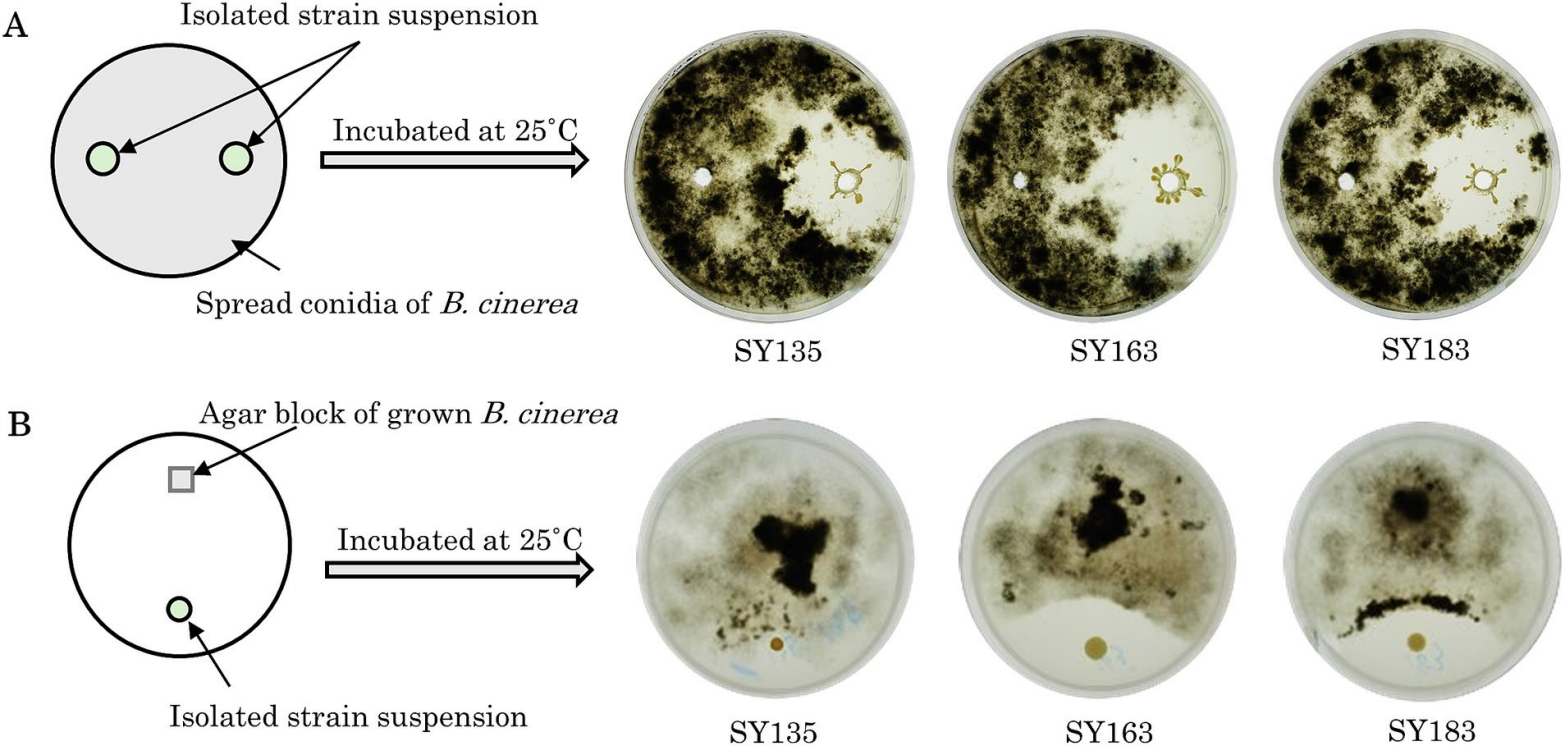
Inhibitory activity of isolates against *Botrytis cinerea*. **(A)** Evaluation using the agar well diffusion method. **(B)** Evaluation using the dual plate assay.

Growth-inhibiting strains were selected based on their ability to inhibit the spread of *B. cinerea* mycelia on the PDA plate. The antifungal activity of 405 isolated strains was evaluated on PDA medium coated with *B. cinerea* suspension using the agar well diffusion method (Figure 3A). If the strain has a growth inhibitory effect on *B. cinerea*, a distinct zone, known as the inhibition zone, is formed around the hole. Many plates were entirely covered with *B. cinerea* mycelium. All 405 strains were tested, and 7 strains exhibited a zone of inhibition against *B. cinerea* (SY50, SY89, SY131, SY135, SY163, SY183, and SY339). Among the strains with confirmed zones of inhibition, SY163 had the largest zone of inhibition (50 mm), followed by SY183 (40 mm) and SY135 (37 mm); the other four strains had smaller zones.

The effects of the seven inhibitory strains on the growth of *B. cinerea* mycelia were examined using the dual plate assay, in accordance with the procedure shown in Figure 3B. The plates were incubated at 25°C for 12 days. All strains exhibited inhibition zones; however, the size of the inhibition zone varied for each strain. The inhibitory rates ranged from 11.9 to 35.7%. A similar trend was observed for the fungus *Metarhizium anisopliae*, which inhibited the radial growth of *B. cinerea* by approximately 43.9% compared with the control (Sarven et al., 2020). The inhibition zone was formed without any contact between *B. cinerea* and the inhibitory strain, suggesting that the inhibitory strain secretes substances that inhibit the growth of *B. cinerea*. Other microorganisms that have been reported to exhibit antifungal activity against *B. cinerea* belong to the genera *Pseudomonas* and *Pantoea* (Trotel-Aziz et al., 2008; South et al., 2020). For example, South et al. conducted a dual plate assay (referred to as a “dual-culture assay” in the cited literature) and placed *B. cinerea* mycelia grown on PDA at the center of a PDA plate. They placed isolates in parallel on either side of the PDA plate to examine the inhibition of *B. cinerea* growth. Several strains exhibited inhibition zones, including *Pseudomonas protegens* AP54, *Pseudomonas chlororaphis* 14B11, and *Pseudomonas fluorescens* 89F1. The formation of such inhibition zones may be due to the production of antibiotics, enzymes, and volatiles by microorganisms (Kerr, 1999; South et al., 2020). Inhibition zones were also observed for the seven inhibitory strains, suggesting that the growth of *B. cinerea* was inhibited by the products of these strains.

**Figure 4:**
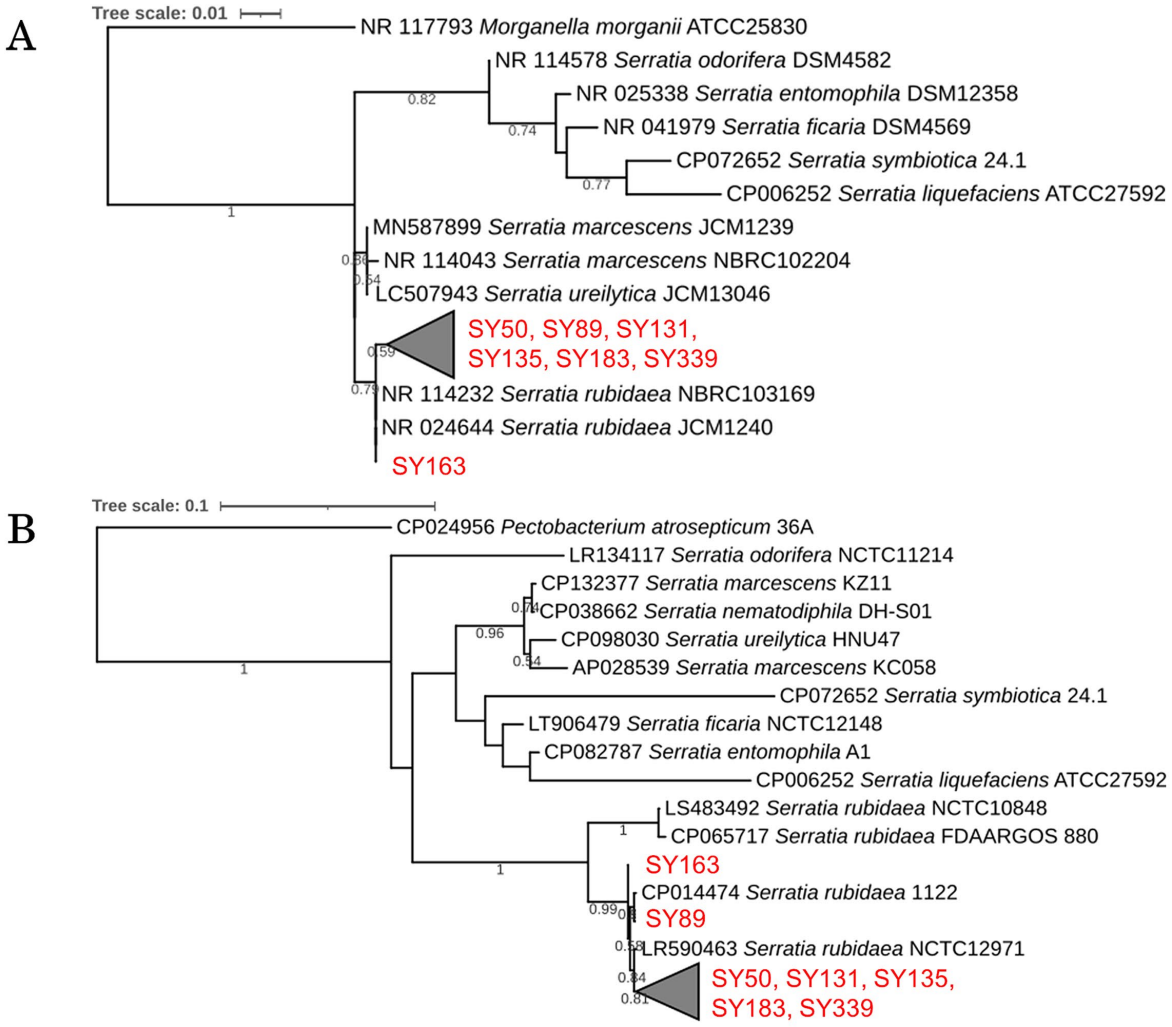
Phylogenetic analysis of inhibitory strains. (A) Phylogenetic tree based on 16S rRNA gene sequences of the inhibitory strains, showing their relationship with known species. (B) Phylogenetic tree based on *gyrB* gene sequences, highlighting genetic differences among the strains.

A homology search targeting 16S rRNA gene sequences was performed for the seven strains that showed antifungal activity against *B. cinerea*; the results are presented as a phylogenetic tree (Figure 4A). All seven strains were approximately 100% or 99% identical to *Serratia rubidaea* JCM1240 and *S. rubidaea* NBRC103169 and were distinct from the strains *S. marcescens* and *S. ureilytica*. Based on these results, all seven strains were closely related to *S. rubidaea*.

A homology search targeting *gyrB* was performed for the seven strains (SY50, SY89, SY131, SY135, SY163, SY183, and SY339) that showed antibacterial activity against *B. cinerea*; their phylogenetic trees are presented in Figure 4B. Strains SY50, SY131, SY135, SY183, and SY339 differed by 1 bp. These five strains (1,171 bp in length) differed from SY89 by 7 bp, and SY163 had a substitution of 4 bp. In addition, SY89 and SY163 strains differed by 5 bp. Consequently, the obtained inhibitory strains could be divided into three groups: (1) SY163, (2) SY89, and (3) SY50, SY131, SY135, SY183, and SY339.

The seven strains that exerted growth inhibitory effects on *B. cinerea* belonged to the genus *Serratia* and were detected in four of the six samples in microbial community analysis. The relative abundance of *Serratia* in each sample was as follows: S1a (0.82%), S1b (1.02%), S3a (0.38%), and S3b (0.33%). The genus *Serratia* included sequences classified as *S. rubidaea* at the NGS species level.

### 3.4 Biological control activity of bacterial isolates in plants

Grafted tomato saplings were grown in a plastic greenhouse in the laboratory and watered regularly. The saplings were administered with only water for 22 days at room temperature (18°C–22°C); these saplings grew without developing diseases, including gray mold, on the leaves. This growth method was confirmed to be suitable for evaluating biocontrol activity.

**Figure 5:**
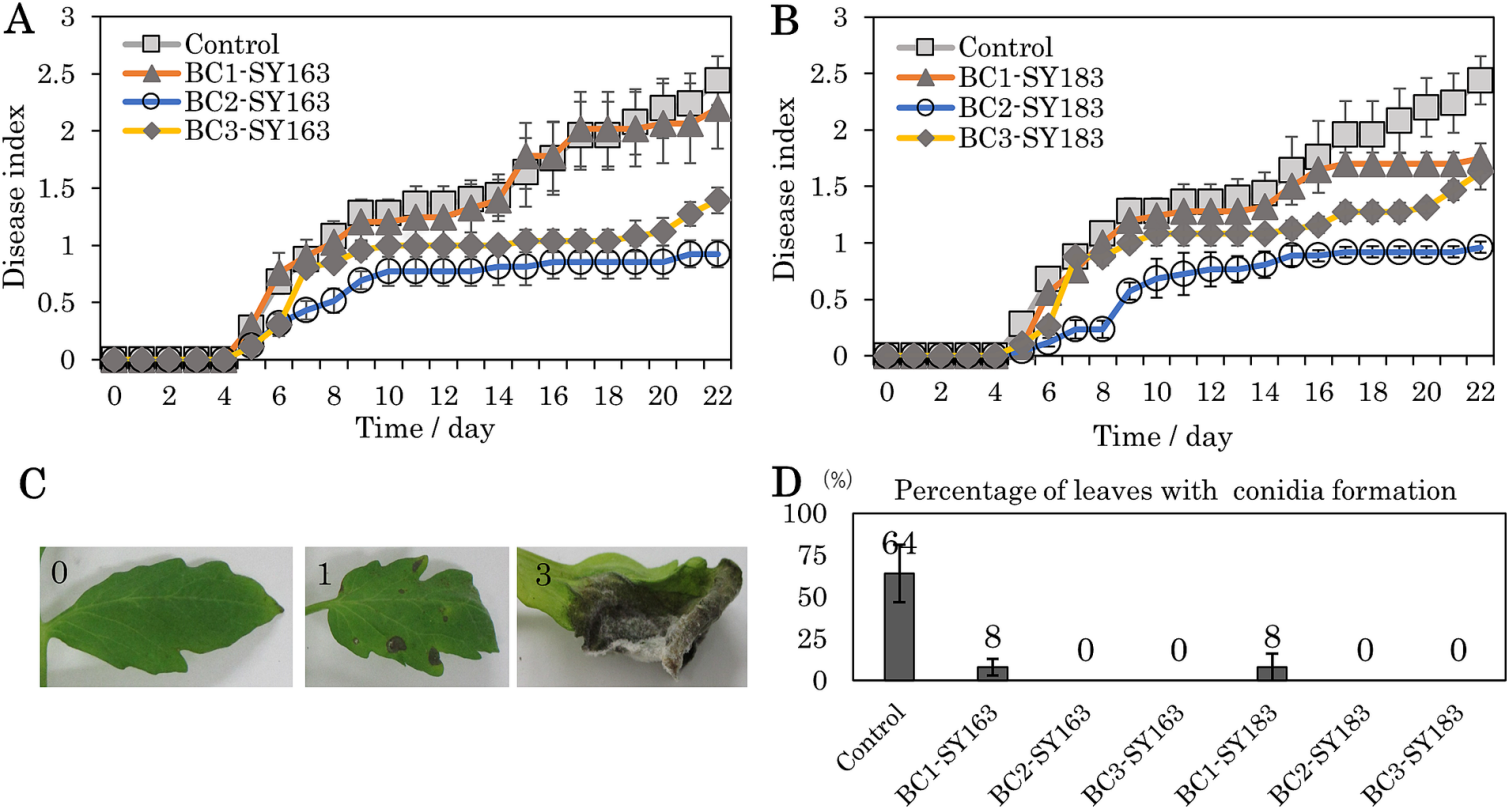
Effects of inhibitory strains on the development of gray mold in tomato plants. (A,B) Changes in the disease index (A: SY163 application, B: SY183 application). Each point and error bar represent the mean and standard error of the disease severity score. (C) Photographs of typical leaves evaluated with a disease index of 0, 1, or 3 (0 = no disease symptoms, 1 = 0.1–24.9%, 3 = 50–100%). Many leaves with a disease index of 3 showed conidia formation. (D) Mean percentage of leaves with conidia formation after 22 days. Each error bar indicates standard error. Conditions: Control, Only *B. cinerea* applied on day 0; BC1 (BC1-SY163, BC1-SY183), *B. cinerea* and each inhibitory strain (SY163 or SY183) applied on day 0; BC2 (BC2-SY163, BC2-SY183), each inhibitory strain applied on the day before day 0, *B. cinerea* and each inhibitory strain applied on day 0; BC3 (BC3-SY163, BC3-SY183), *B. cinerea* and each inhibitory strain applied on day 0 and each inhibitory strain applied on day 1.

The inhibitory effect of bacterial strains on gray mold suppression in tomato leaves was investigated. Two inhibitory strains, SY163 and SY183, which showed significant differences in *gyrB* sequence, were applied onto plants, and the disease index was evaluated on a scale of 0–3 (Figure 5C). The 22-day disease index results are shown in Figures 5A,B. In the control group, *B. cinerea* was sprayed onto tomato leaves on day 0, and disease development started on day 5. Disease index scores up to day 22 are presented in Figures 5A,B. The disease index score gradually increased to >2 on day 22. Under condition BC1, *B. cinerea*, and strain SY163 (BC1-SY163) or SY183 (BC1-SY183) were applied on day 0. Compared with the control group, there was lower disease suppression in BC1-SY163, but a trend toward further disease suppression was observed in BC1-SY183. Under condition BC3, *B. cinerea* and strain SY163 (BC3-SY163) or SY183 (BC3-SY183) were applied on day 0, and strain SY163 or SY183 was applied again on day 1. In BC3-SY163 and BC3-SY183, higher disease suppression was observed compared with the control and BC1 groups, respectively. Under condition BC2, two inhibitory strains (SY163 and SY183) were each applied on the day before day 0, and *B. cinerea* and the inhibitory strains were applied and allowed to grow on day 0. Under all conditions, application of the inhibitory strains reduced the disease index of gray mold. For both SY163 and SY183 strains, the disease index tended to be lower under condition BC2 than under control group or conditions BC1 and BC3.

To compare the disease index between conditions, AUDPC values were calculated for all samples. The means and standard errors for each condition are shown in Table 1. Both SY163 and SY183 showed the smallest AUDPC values under condition BC2. When comparing the mean AUDPCs of BC1-SY163, BC2-SY163, and BC3-SY163 against the mean AUDPC of control group using analysis of variance and Dunnett test, a statistically significant difference was observed between control group and BC2-SY163 (*p* < 0.01). When comparing the mean AUDPCs of BC1-SY183, BC2-SY183, and BC3-SY183 against the mean AUDPC of control group, a statistically significant difference was noted between control group and BC2-SY183 (*p* < 0.001) and between control group and BC3-SY183 (*p* < 0.05).

**Table 1:** Comparison of the area under the disease progression curve (AUDPC) for each condition.

Condition	Control	BC1-SY163	BC2-SY163	BC3-SY163	BC1-SY183	BC2-SY183	BC3-SY183
AUDPC (mean ± SE)	26.1 ± 2.9	25.5 ± 4.0	12.5 ± 1.9**	16.4 ± 1.4	22.5 ± 0.74	11.8 ± 1.4***	18.2 ± 1.1*

Asterisks indicate statistically significant differences in the AUDPC for each condition relative to A (*p < 0.05, **p < 0.01, ***p < 0.001, ANOVA, Dunnett’s *post-hoc* test). Control (Control): Only *B. cinerea* applied on day 0; condition BC1 (BC1-SY163, BC1-SY183), *B. cinerea* and each inhibitory strain (SY163 or SY183) applied on day 0; condition BC2 (BC2-SY163, BC2-SY183), each inhibitory strain applied on the day before day 0, *B. cinerea* and each inhibitory strain applied on day 0; condition BC3 (BC3-SY163, BC3-SY183), *B. cinerea* and each inhibitory strain applied on day 0 and each inhibitory strain applied again on day 1.

A linear mixed model was used to evaluate the impact of days and conditions on the disease index. Days and conditions were modeled as fixed effects and individuals (ID) as random effects. The restricted maximum likelihood was used to estimate the parameters of the model. The mean values for each condition estimated from the model were calculated, and Dunnett’s test was used to perform multiple comparisons across conditions. Statistically significant differences were observed between control group and BC2-SY163 (*p* < 0.01), control group and BC2-SY183 (*p* < 0.001), and control group and BC3-SY183 (*p* < 0.05). It was suggested that spraying the strain SY163 before *B. cinerea* attachment or the strain SY183 before or at the same time as *B. cinerea* attachment may effectively prevent gray mold.

In addition to differences in the disease index, we evaluated the proportions of leaves forming conidia. As the severity of gray mold increased, conidia formation occurred, as indicated by the score of 3 (Figure 5C). After observation for 22 days, the number of leaves that formed conidia was examined, and the proportion of leaves that formed conidia in the assessed leaves in each pot was calculated. The mean and standard error of the proportion of conidia formation were determined for each condition (Figure 5D). Compared with control group, in which only *B. cinerea* was applied, conidia formation was significantly inhibited under all conditions in which SY163 or SY183 was applied. These two strains of *Serratia* sp. were found to be effective in suppressing the sporulation of *B. cinerea*.

## 4 Discussion

The microbial community analysis of the phylum and genus on the surface of tomato leaves and stems was conducted in this study. Relative abundances higher than 0.5% were found in seven phyla, including *Proteobacteria*, *Firmicutes*, and *Bacteroidetes*. Relative abundances higher than 0.5% were observed in 54 genera, including *Lactococcus, Lactobacillus, Enterobacter, Pantoea, Mycoplasma*, *Marinomonas*, and *Enterobacter*. The microbial community structures of samples collected from the same location were similar; however, differences were observed between samples collected from different locations. Previous studies have described the symbiotic microbial communities of various plants. Compared with the microbial community analysis of endophytic and phylloplane bacteria in tomato leaves using milled samples (Sumbula et al., 2020), this study also identified *Proteobacteria*, *Bacteroidetes*, and *Actinobacteria* at the phylum level and *Acinetobacter*, *Bacteroides*, and *Pantoea* at the genus level, with certain relative abundances. Compared with the microbial community analysis of phylloplane bacteria in rice (Sumbula et al., 2020), this study also identified *Proteobacteria*, *Actinobacteria*, *Bacteriodetes*, and *Firmicutes* at the phylum level and *Methylobacterium* and *Serratia* at the genus level, with certain relative abundances. The phyllosphere microbial community can vary depending on plant species, geographic location, season, and human intervention (Knief et al., 2012; Sumbula et al., 2020). In this study, the microbial communities identified on tomato leaves and stems also varied depending on the collection site, thereby supporting these findings. Microbial community analysis of tomato leaves and stems identified 13 genera containing methylotroph strains. For example, 80 isolates were obtained from peanut leaf surface samples (Krishnamoorthy et al., 2018) via methanol-containing cultures and were identified. These isolates were classified into 15 genera, with *Pantoea*, *Pseudomonas*, *Bacillus*, and *Acinetobacter* commonly identified with this study. The genus diversity of methylotrophs also varied depending on the collection site, thereby supporting the results of this study. Methylotroph isolates have multiple properties that promote plant growth, including production of indole-3-acetic acid (IAA), siderophores, and 1-aminocyclopropane-1-carboxylate deaminase, nitrogen fixation, sulfur oxidation, and solubilization of insoluble minerals. Other studies have also reported IAA production and nitrogen fixation in methylotrophs (Senthilkumar et al., 2009; Madhaiyan et al., 2015). These findings suggest that diverse methylotroph promote plant growth through various mechanisms, which might be the case for tomato leaves and stems in this study.

In this study, plant surface samples were cultured on a medium containing methanol as the single carbon source, and *S. rubidaea* strains were selected as strains with antifungal activity against *B. cinerea*. The genus *Serratia* has rarely been reported as a methylotroph. However, it is possible that *Serratia* grows in methanol and that it was cultured and isolated in this study on a methanol-containing medium due to co-cultivation with methylotrophs. These strains have the potential to act as PGPB as well as biocontrols. Previous reports on PGPB belonging to the genus *Serratia* demonstrated that *S. liquefaciens* KM4 may promote plant growth and improve salt stress tolerance by regulating ion homeostasis, leaf gas exchange, and expression of stress-related genes (El-Esawi et al., 2018). *S. marcescens* strains reportedly act as PGPBs, as evidenced by the production of various lytic enzymes (proteases, lipases, cellulases, and catalases), antimicrobial compounds (hydrogen cyanide and siderophores), ammonia, nitrite, and nitrate as well as their ability to reduce nitrate to nitrite (Hamada and Soliman, 2023). Recently, a strain of *S. rubidaea* was reported to promote the germination of quinoa seeds in salt-tolerant environments (Mahdi et al., 2021). Another strain of *S. rubidaea* promoted potato growth (Hanifah et al., 2023). Therefore, it was expected that *S. rubidaea* strains in this study may act as PGPB.

Various antifungal substances have been known to be produced from *Serratia* strains. *S. liquefaciens* strains collected from grape leaves and *S. marcescens* B2 strains collected from tomato leaves have been reported to inhibit mycelial growth of *B. cinerea* (Akutsu et al., 1993; Whiteman and Stewart, 1998). Chitinase from *Serratia* spp. may attack chitin, a major component of fungal cell walls, causing its degradation and the death of pathogens (Singh and Jha, 2016). *S. rubidaea* MarR61-01, isolated from healthy strawberry stems, has shown antifungal activity against *B. cinerea in vitro* (Alijani et al., 2023). In addition to the involvement of prodigiosin produced by *S. rubidaea* MarR61-01 in the suppression of *B. cinerea* growth, multiple pathways have been proposed. Moreover, VOCs produced by bacteria and plant defense responses may contribute to plant pathogen control. Although the growth inhibitor of *B. cinerea* produced by the *S. rubidaea* strain was not identified, it is possible that the mechanism of action may be similar to that underlying the antifungal activity of these analogous bacteria.

Regarding biosafety, some *S. rubidaea* strains have been obtained from human foci. Recently, some *Serratia* strains showed potential biocontrol activity against plant and human pathogens (Alijani et al., 2023). For example, regarding *Serratia marcescens*, there has been a discussion about the selection of strains that cause human disease and those that are safe as PGPB (Abreo and Altier, 2019). Given the variety of populations and novel species within *S. marcescens*, research is underway to isolate opportunistic pathogenic strains or those with potential for agricultural use (Abreo and Altier, 2019; Cho et al., 2020). In addition, safety information is necessary when handling *S. rubidaea* strains in fields. However, this study provides fundamental knowledge for developing techniques to control the relative abundance of *S. rubidaea* strains endemic to plants.

Previous studies have demonstrated the effectiveness of various microbial agents in controlling *B. cinerea*. For example, culture supernatants of *Metarhizium anisopliae* or *Pseudomonas* strain QBA5 were applied to detached tomato leaves and post-harvest tomato fruits prior to *B. cinerea* application. In these studies, both *Metarhizium anisopliae* and *Pseudomonas* strain QBA5 were effective in controlling *B. cinerea* (Sarven et al., 2020; Gao et al., 2018). Another study applied a cultured bacterial suspension of *Pseudomonas aeruginosa* CQ-4 to detached leaves and greenhouse-potted tomato plants prior to the application of *B. cinerea*. The concentration of conidia solution was 1.0 × 10^6^ CFU/mL for *B. cinerea* and 1.0 × 10^8^ CFU/mL for *P. aeruginosa* CQ-4. The results demonstrated that the *P. aeruginosa* CQ-4 strain inhibited the growth of *B. cinerea* (Wang et al., 2021). Evaluation of disease development after 20 days showed that the application of *Pseudomonas aeruginosa* CQ-4 suppressed gray mold. In cyclamen, disease suppression has been reported with *S. marcescens* strain B2 (Iyozumi et al., 1996). After cyclamen petals were sprayed with *S. marcescens* B2 (10 mL of *ca.* 1 × 10^9^ CFU/mL per plant), *B. cinerea* (10 mL of *ca.* 5 × 10^5^ conidia/mL per plant) was sprayed and disease development was evaluated. After 2 weeks of growth and condition observation, the disease suppression effect was confirmed. In this study, we evaluated gray mold on grafted tomato seedlings and found that application of suppressant strains at lower concentrations than those reported in previous studies had similar disease suppression effects. In addition, we confirmed the inhibitory effects of *S. rubidaea* strains on *B. cinerea* conidia formation. As conidia formation increases the risk of infection spread by conidia dispersal (Barnes and Shaw, 2003), this may significantly contribute to the observed suppression of gray mold.

In this study, microbial community analysis revealed the presence of the genus *Serratia*, including *S. rubidaea,* in tomato leaf and stem samples and demonstrated that the relative abundance of this genus varied among samples. We also identified the relative abundance of PGPB with confirmed antifungal activity in the microflora of plant surface samples for the first time. Although external application of PGPB can have positive effects on plant growth and pest control, there are concerns regarding the PGPB load on the microbial community, with its effect being transient and weak in certain growing environments (Vuolo et al., 2022). These concerns could be addressed by focusing on indigenous PGPB with antifungal properties and controlling their relative abundance. In the future, regulating the relative abundance of *S. rubidaea* strains with biocontrol properties and maintaining a microflora that promotes plant growth might allow the establishment of a more optimal environment for both plant growth and biological control. This approach aims to leverage the power of nature, paving the way for sustainable agriculture, thereby harmonizing growth enhancement with effective biological control.

## Data Availability

The datasets presented in this study can be found in online repositories. The names of the repository/repositories and accession number(s) can be found at: https://www.ddbj.nig.ac.jp/, accession numbers LC820854-LC820860, LC822350-LC822356 and PRJDB18246.

## Footnotes

^1^ https://qiime2.org/

^2^ https://www.arb-silva.de/

^3^ https://www.r-project.org/

^4^ https://www.r-project.org/
