# Supplementary material for: Selection and application of methanol-utilizing bacteria from tomato leaves for biocontrol of gray mold
Source: Front Microbiol. 2024 Oct 18;15:1455699. doi: 10.3389/fmicb.2024.1455699 (PMC11527628; doi:10.3389/fmicb.2024.1455699)
Supplement: Supplementary file 1 [file Data_Sheet_1.PDF]

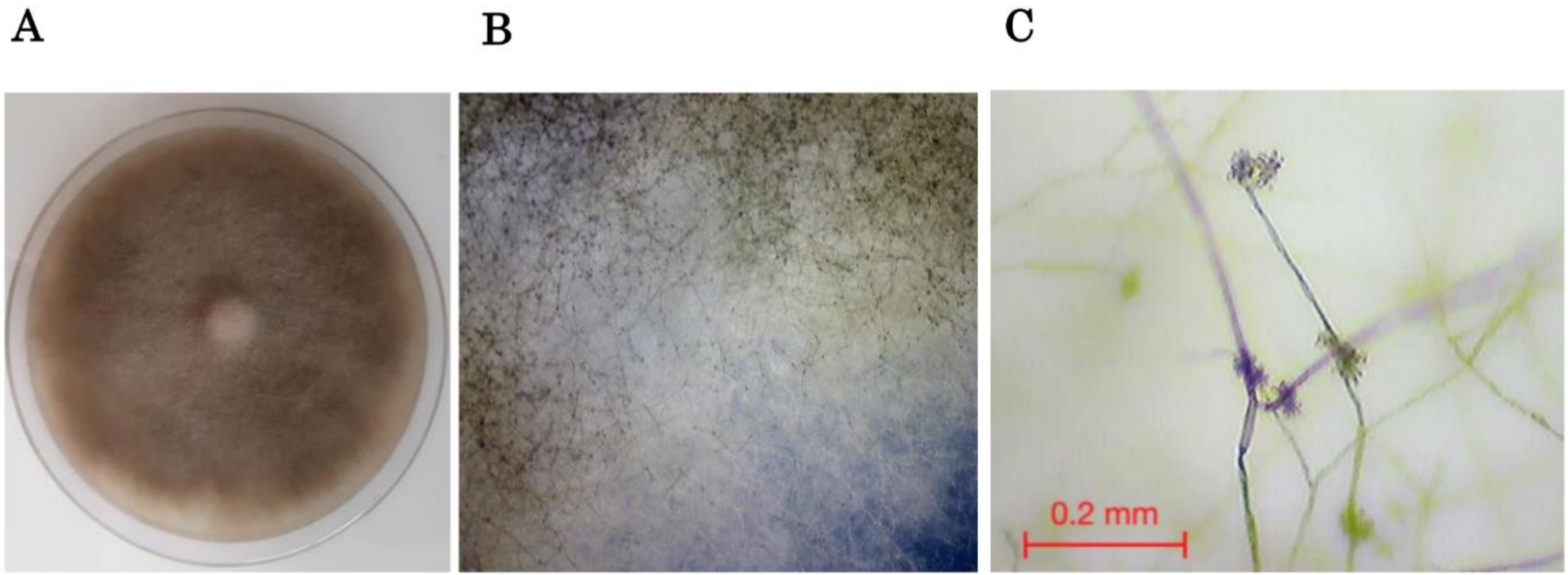

Figure S1. *Botrytis cinerea* culture on PDA plates. (A) *B. cinerea* mycelium covering a PDA plate. (B, C) Magnified view of *B. cinerea* mycelium under a stereomicroscope.

Table S1. Alpha diversity of the microbial community in samples of tomato leaves and stems by the observed OTUs, ACE values, and Chao1, Shannon, Simpson indices.

Sample	Reads after filtering	Observed OTUs	Chao1	ACE	Shannon	Simpson
S1a	13894	257	258	257	4.73	0.984
S1b	16091	246	246	246	4.38	0.960
S2a	12751	143	143	143	3.82	0.941
S2b	12354	160	160	160	3.93	0.943
S3a	97048	140	140	141	2.75	0.847
S3b	102195	138	138	138	2.79	0.855

Table S2. Relative abundances (%) of the genus containing methylotrophs in tomato leaves and stems.

	S1a	S1b	S2a	S2b	S3a	S3b
Pantoea	1.60	1.19	12.66	11.87	3.84	3.56
Pseudomonas	4.18	2.90	4.29	4.67	0.09	0.08
Bacillus	3.33	3.30	1.88	1.37	0.06	0.06
Klebsiella	2.71	1.79	0.35	0.61	0.15	0.16
Acinetobacter	1.38	1.46	0	0.11	0.02	0.01
Bacteroides	0.87	0.74	0	0	0	0
Paracoccus	0.14	0.59	0.47	0.40	0	0
Brevibacterium	0.20	0.05	0.26	0.40	0.10	0.07
Sphingomonas	0	0.18	0	0	0.09	0.07
Mycobacterium	0	0.18	0	0	0.05	0.05
Methylobacterium	0	0.11	0	0.08	0	0
Flavobacterium	0.10	0.09	0	0	0	0
Mesorhizobium	0	0	0	0.09	0	0
Total	14.51	12.58	19.91	19.60	4.40	4.07
